# From Surfaces to Spillover: Environmental Persistence and Indirect Transmission of Influenza A(H3N8) Virus

**DOI:** 10.3390/microorganisms13122782

**Published:** 2025-12-06

**Authors:** Yifei Jin, Huan Cui, Lina Jiang, Li Li, Jing Zheng, Yidun Zhang, Heng Wang, Yanrui Li, Yan Wang, Yixin Zhao, Cheng Zhang, Zhixin Yang, Yan Zhang, Zhongyi Wang

**Affiliations:** 1 Academy of Military Medical Sciences, Academy of Military Sciences, Beijing 100071, China; christina_jyf@foxmail.com (Y.J.); wangheng946@163.com (H.W.); lyr98@foxmail.com (Y.L.); w1377876707y@foxmail.com (Y.W.); yixinzhao1129@foxmail.com (Y.Z.); yy_xiao@126.com (Z.Y.); 2 College of Veterinary Medicine, Hebei Agricultural University, Baoding 071000, China; cuihuan1349@163.com (H.C.); zc1349@foxmail.com (C.Z.); 3 Xiamen Center for Disease Control and Prevention, Xiamen 361021, China; ln_jiang@126.com (L.J.); fjxmlily@163.com (L.L.); zhengjing1103@foxmail.com (J.Z.); eatonzhang@163.com (Y.Z.)

**Keywords:** influenza A(H3N8) virus, environmental persistence, indirect transmission, live poultry market, infection risk assessment

## Abstract

Avian influenza viruses (AIVs) pose a significant zoonotic threat, with the emerging H3N8 subtype raising increasing concern due to sporadic human infections. Current strategies for risk assessment of novel AIVs primarily rely on genetic surveillance and isolated case reports, which provide limited insight into their cross-species transmission potential. However, these approaches may overlook critical phenotypic determinants, such as pathogenicity, transmissibility, and environmental persistence, that directly influence zoonotic risk. This study investigates the evolutionary relationships, receptor-binding properties, replication dynamics, pathogenicity in mice, transmission efficiency in guinea pigs, and environmental persistence of three H3N8 strains isolated from a live poultry market. All three H3N8 strains bound exclusively to α-2,3 sialic acid receptor and achieved 100% transmissibility among guinea pigs through direct contact. Notably, the environment-origin strain A09 exhibited an indirect contact transmission efficiency of 33.3%. The findings reveal strain-specific differences, with A09 displaying enhanced pathogenicity, broader transmission routes, and greater environmental persistence compared with A05 and A01. This perspective underscores the value of integrated profiling from genotype to phenotype combined with multi-route transmission and environmental persistence analyses to delineate the adaptive roadmap of H3N8 from avian to mammalian hosts and to assess its emerging infection risk. Future directions for surveillance and intervention were also discussed, highlighting their potential to strengthen preparedness against zoonotic influenza threats.

## 1. Introduction

The transmission of avian influenza virus (AIV) among mammals typically occurs through three primary routes: direct contact transmission, airborne transmission, and indirect contact transmission. Indirect contact transmission refers to the infection of susceptible individuals or animals through exposure to contaminated items harboring the virus from infected hosts. This mode of transmission commonly occurs in environments characterized by close human–poultry interactions, such as live poultry markets (LPMs), farms, and slaughterhouses [1,2,3]. Previous human cases of H3N8 AIV infection reported in China were associated with exposure to contaminated environments [4,5]. These findings indicate that shared environments between humans and poultry serve as key initial sites for cross-species transmission of AIV and play a foundational role in the virus’s ability to overcome species barriers [6]. Therefore, investigating the evolutionary dynamics of indirect transmission efficiency of AIVs in mammalian hosts within close human–poultry contact environments is of paramount importance.

Environmental persistence is a critical factor influencing the replication, dissemination, and inactivation of AIVs in LPMs [7]. Environmental surveillance plays a crucial role in assessing the risk of avian influenza transmission [8]. As indirect contact via contaminated environments represents an important transmission route, monitoring viral persistence on different surfaces and under varying conditions provides valuable insight into environmental persistence and pathogen characteristics [9,10,11]. Longitudinal surveillance in LPMs revealed that temperature and relative humidity significantly affecting viral survival [1,12,13,14]. These findings underscore that environmental persistence can create a temporal window for indirect transmission, highlighting the importance of targeted environmental monitoring and disinfection strategies in high-risk areas. Strengthening such surveillance can improve our understanding of viral adaptation, enhance early warning systems, and ultimately contribute to more effective prevention and control strategies [15]. Key tolerated environmental factors include temperature, humidity, and surface materials [16,17,18]. Therefore, this study focuses on the indirect contact transmission potential and environmental persistence of the H3N8 AIV, aiming to identify direct correlative evidence between these two aspects. The surface materials selected for this investigation—plastic, glass, fabric, and stainless steel—among the most commonly encountered materials in daily operations within LPMs. Furthermore, due to the culinary preference in southern China for freshly slaughtered poultry, this region represents a high-risk area for human infection with AIVs in such markets. Accordingly, this study examines temperature gradients commonly found across seasons in southern China, including 4 °C, 25 °C, and 37 °C.

**Scheme 1:**
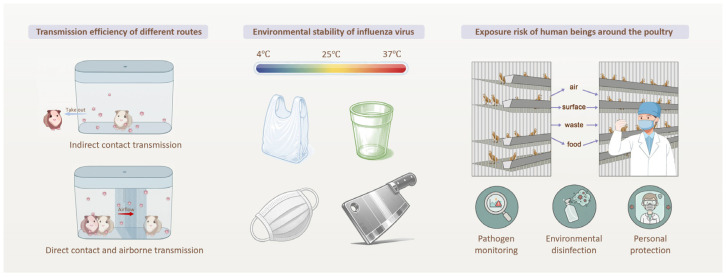
Schematic illustration of transmission efficiency, environmental persistence, and human exposure risk of H3N8 avian influenza viruses. Left: Transmission efficiency of different routes in guinea pigs, including direct contact, airborne, and indirect contact transmission. Middle: Environmental persistence of influenza virus on common materials (plastic, glass, fabric, stainless steel) under three representative temperatures (4 °C, 25 °C, and 37 °C). Right: Exposure risk of humans in live poultry markets, where potential transmission routes include contaminated air, surfaces, waste, and food. Strategies for reducing infection risk include pathogen monitoring, environmental disinfection, and personal protection.

Despite increasing concern about the zoonotic potential of H3N8, systematic studies evaluating its indirect transmission efficiency in mammalian hosts and its environmental persistence under realistic market conditions remain scarce. Previous reports have largely focused on genetic characterization or isolated human cases, providing limited insights into the interplay between viral evolution, host adaptation, and environmental persistence. In addition, AIVs can remain infectious on a variety of surfaces for hours to days, with survival strongly influenced by material type, temperature, and humidity [12,19,20,21]. These studies suggest that environmental reservoirs, particularly in live poultry markets or other high-contact settings, may facilitate indirect transmission. However, direct experimental evidence linking environmental persistence to actual transmission efficiency remains limited. In this study, we integrated profiling from genotype to phenotype to predict adaptive roadmap of H3N8 from avian to mammalian host and evaluated multi-route transmission efficiency and environmental persistence to assess the infection risk of emerging H3N8 (Scheme 1). This combined approach provides novel evidence linking environmental persistence with transmission efficiency, thereby offering critical insights into the mechanisms underlying cross-species transmission risk of emerging H3N8 avian influenza viruses.

## 2. Materials and Methods

### 2.1. Viruses

In January 2022, three strains of H3N8 AIV were isolated and cultured from chicken feces, cage surface samples, and wastewater from poultry cleaning at a LPM in Xiang’an District, Xiamen City, China. These strains were designated as A/Chicken/Xiamen/A05/2022(H3N8), A/Environment/Xiamen/A09/2022(H3N8), and A/Environment/Xiamen/A01/2022(H3N8). These three strains are, respectively, abbreviated as A05, A09 and A01.

### 2.2. Genome Sequencing and Phylogenetic Analysis

Viral RNAs were extracted from HA-positive allantoic fluid by using QIAamp Viral RNA Mini Kit (QIAGEN, Hilden, Germany). Viral RNAs were reverse-transcribed into cDNA by using the primer Uni12 and RT reagent Kit (Takara Bio, Otsu, Japan). The obtained cDNA was used for specific amplification and viral genome sequencing (Sangon Biotech (Shanghai) Co., Ltd., Shanghai, China). All reference sequences used in this study were obtained from the National Center for Biotechnology Information GenBank database, and then the downloaded sequences were compared with the strains in this study through Cluster W. The GTRGAMMA nucleotide substitution model in PhyML 3.1 software was used and bootstrap replicates were run 1000 to evaluate the Maximum likelihood (ML) phylogenies of codon comparison between the two gene sequences. The phylogenetic relationship of the HA gene of four strains was inferred with BEAST v1.10.4 by using a molecular clock that placed a timescale on virus evolution to estimate rates of viral evolution and dates of divergence13. Phylogeny is estimated within the framework of Bayesian Markov chain Monte Carlo (MCMC). A maximum clade credibility (MCC) tree with mean height was generated for each dataset by using TreeAnnotator version 1.10.4 (https://beast.community/treeannotator, accessed on 1 October 2025) after 10% burn-in. In addition, at least two independent runs were carried out and compared to ensure sufficient sampling. Phylogenetic tree and MCC tree were visualized by using FigTree version 1.4.4 (http://tree.bio.ed.ac.uk).

### 2.3. Receptor-Binding Specificity Identification

To detect the receptor-binding specificities of four strains, we performed HA assays with 1% chicken red blood cells (cRBCs) suspension. Sialic acid in chicken erythrocytes was treated with Vibrio cholerae neuraminidase (VCNA, Roche, San Francisco, CA, USA) at 37 °C for 1 h. After the VCNA is processed, part of suspension was treated with α-2,6-(N)-sialyltransferase or α-2,3-(N)-sialyltransferase (Sigma-Aldrich) to cRBCs at 37 °C for 3 h. HA assays were performed using 4 different chicken erythrocytes obtained above to analyze the receptor binding specificity of different strains. The poultry-isolate A/chicken/Hebei/HB777/2006 (HB777[H5N1]) and human-isolate A/California/04/2009 (CA04[H1N1]) viruses were used as controls for preferential binding to avian-type SAα-2,3-Gal and human-type SAα-2,6-Gal, respectively.

### 2.4. Growth Dynamics in Cells

The growth kinetics of four strains in A549 and MDCK cells were compared according to the protocol of previous study [1]. Each strain was inoculated into monolayers of A549 or MDCK cells at a multiple of infection (MOI) of 0.001. Supernatants of infected cells were collected at 12, 24, 36, 48, 60, and 72 h post infection and stored at −80 °C, and the virus titer of each collected sample was determined by EID_50_ assays.

### 2.5. Mouse Experiments

The pathogenicity of each H3N8 strain in mammals was evaluated by mouse experiments. Six-week-old BALB/c female mice (Vital River Laboratory Animal Technology Co., Ltd., Beijing, China) were used in this study. The mice infection experiments were divided into two parts. The first part is weight monitoring, and the second part is the monitoring of virus replication and pathogenicity in vivo. Viral growth in each tissue was determined by EID_50_ assays. H&E staining of mice lung were performed 3 days post inoculation. Pathological severity scores in infected mice, based on the inflammation area percentage for each section of the lungs collected from each animal using the following scoring system: 0, no pathological change; 1, affected area ≤ 10%; 2, affected area <50% and >10%; and 3, affected area ≥ 50%; an additional point was added to the score when pulmonary edema and/or alveolar hemorrhage was observed. Lung Index = (W1/W0) × 100 where W1 is the lung wet weight (g); W0 is the body weight (g). This index was used to assess the degree of pulmonary inflammation and edema induced by viral infection.

### 2.6. Assessment of Viral Environmental Persistence

The experiment involved applying each virus to four different materials (plastic, glass, fabric, and stainless steel), followed by incubation at three different temperatures (4 °C, 25 °C, and 37 °C) and 50% relative humidity. Measurements were taken at five time points (0.5 h, 1, 3, 5, and 7 days) for each temperature. The stock AIV solution was diluted to 10^6^ EID_50_/mL with PBS (containing penicillin and streptomycin), and 1 mL of the diluted working solution was applied to the surface of each material. After being loaded with virus, the materials were incubated at various temperatures. At specific time points, 1 mL of PBS was added to each material and pipetted repeatedly to recover residual virus from the surface. If the virus suspension had not dried, quantification to 1 mL was performed before recovery. Recovered samples underwent virus titer (EID_50_) determination using SPF chicken embryos, assessing the persistence of H3N8 on different materials under diverse temperature conditions. Five replicates were set up for each virus.

### 2.7. Transmission Experiments Among Guinea Pigs

For direct contact transmission, one donor guinea pig at 24 h post inoculation was placed together with one naïve guinea pig at the same time for 7 days. For airborne transmission, one donor guinea pig at 24 h post inoculation and another naïve guinea pig was placed in adjacent cages at the same time for 7 days, separated by 5 cm, without direct physical contact with the infected donor. For indirect contact transmission, one donor guinea pig at 24 h post inoculation was housed in a clean cage, then the donor guinea pig was removed after another 24 h and one naïve guinea pigs was subsequently introduced into the same cage for 7 days to assess transmission through contaminated environments. All experiments were conducted with six independent biological replicates. Nasal wash samples were collected on 1, 3, 5 and 7 days post inoculation. Serum was collected from all guinea pigs 21 days post inoculation and the HI test was performed according to the protocol described in the World Health Organization guidelines.

### 2.8. Statistical Analysis

One-way analysis of variance (ANOVA) was performed using GraphPad Prism 8.0 to determine statistically significant differences. All analyses were performed in triplicate and are representative of at least three separate experiments. Error bars represent standard deviation.

## 3. Results

### 3.1. Genotype-to-Phenotype Insights into H3N8 Adaptation of Cross-Species Infection

**Figure 1:**
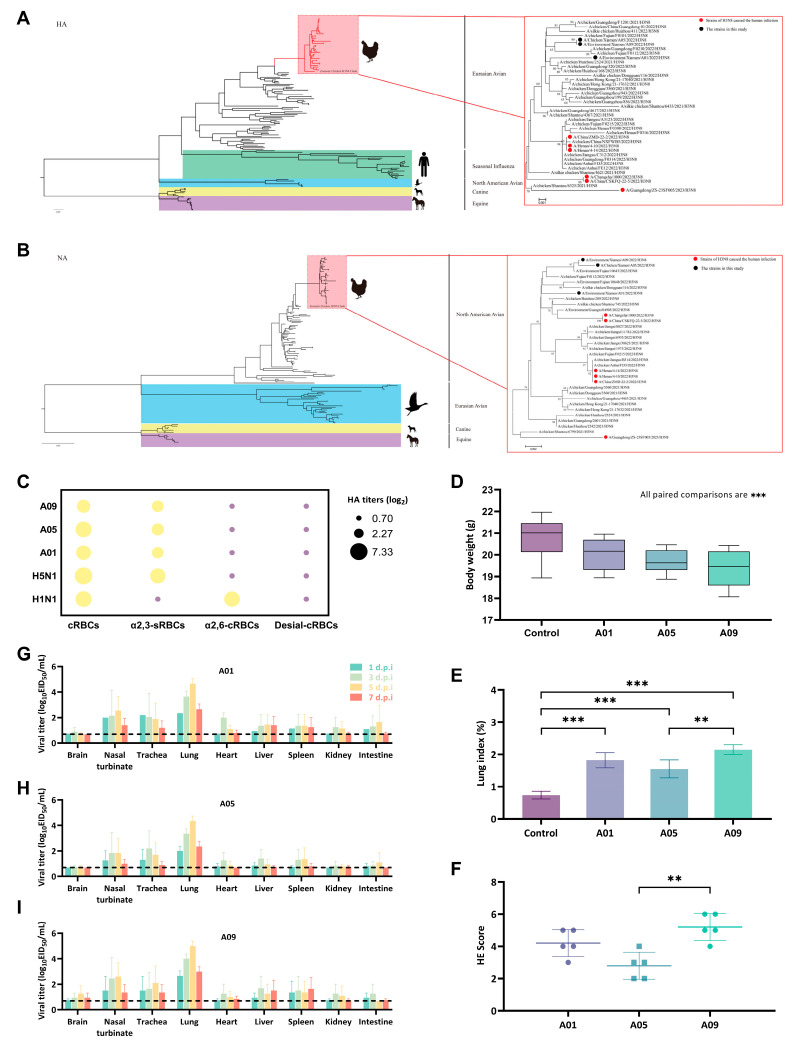
Integrated profiling from genotype to phenotype predicts adaptive roadmap of H3N8 from avian to mammalian host. (**A**,**B**) show the phylogenetic tree of the hemagglutinin (HA) and neuraminidase (NA) genes of H3N8 viruses. The black dots represent the isolated H3N8 strains in this experiment, while the red dots represent the H3N8 human infection strains. (**C**) Receptor binding property of three H3N8 strains. Purple indicates a negative result, and yellow indicates a certain binding ability; the larger the circle, the stronger the binding ability. (**D**) Bodyweight change of mice after inoculation with different strains. *** *p* < 0.0005. (**E**) Lung coefficient of mice after inoculation with different strains. ** *p* < 0.005, *** *p* < 0.0005. (**F**) Pathological severity scores from H&E staining of mice lung sections, based on the inflammation area percentage for each section of the lungs collected from each animal using the following scoring system: 0, no pathological change; 1, affected area ≤ 10%; 2, affected area < 50% and >10%; and 3, affected area ≥ 50%; an additional point was added to the score when pulmonary edema and/or alveolar hemorrhage was observed. ** *p* < 0.005. (**G**–**I**) Viral titers in nine organs (brain, nasal turbinate, trachea, lung, heart, liver, spleen, kidney and intestine) of mice that were infected with A01 (**G**), A05 (**H**) and A09 (**I**) on 1, 3, 5, and 7 days post inoculation. The dashed lines represent the minimum detection limit.

The results of integrated profiling from genotype to phenotype are shown in Figure 1. ML phylogenetic analysis of the HA gene showed that all three H3N8 isolates obtained in this study clustered within the Eurasian avian lineage, with the closest genetic relationship observed between strains A05 and A09 (Figure 1A). Similarly, ML analysis of the NA gene revealed that these isolates belonged to the North American avian lineage, again showing that A05 and A09 were most closely related (Figure 1B). Although the three H3N8 isolates obtained in this study clustered within the same lineage as six previously reported human-derived H3N8 viruses in both the HA and NA genes, they remain more closely related to avian-origin H3N8 strains. The receptor binding assay results show that the control group strains H5N1 and H1N1, respectively, have α-2,3 or α-2,6 sialic acid receptor binding properties; the 3 strains of H3N8 AIVs in the experimental group can all bind to the α-2,3 sialic acid receptor (Figure 1C). The cell proliferation assay showed that in MDCK cells, all three H3N8 strains showed similar replication kinetics, with viral titers steadily increasing and reached their peak proliferation at 72 h (Figure S1). Notably, the replication level of the avian-origin A01 strain was slightly lower compared with the environmental-origin A01 and A09 strains. In A549 cells, the A09 strain exhibited a slightly faster growth trend, reaching its peak proliferation at 60 h, whereas A01 and A05 continued to increase until 72 h (Figure S1). Overall, the replication efficiency of the three strains in both MDCK and A549 cells was comparable, with A09 showing a modest growth advantage in human cells.

Phenotypic validation in mice was performed following H3N8 infection. The body weight of the inoculated mice was monitored daily for 14 days (Figure 1D). All infected animals exhibited varying degrees of weight loss without other apparent clinical symptoms. Among the three H3N8 strains, mice infected with A09 experienced the most pronounced weight reduction, whereas those inoculated with A05 showed the least change. Statistical analysis confirmed significant differences in body weight among the four groups, including the control group (Figure 1D). We further analyzed the phenotype of H3N8-infected mice by assessing the lung index and pathological analysis of lung tissue. The lung index results showed that mice infected with A09 exhibited the highest values, whereas those infected with A05 displayed the lowest among the infected groups. Significant differences were observed between each infected group and the control group, and a notable difference was also detected between the A05- and A09-infected groups (Figure 1E). In the lung tissues of mice inoculated with the three strains, there was a large amount of inflammatory cell infiltration, accompanied by thickening of the alveolar walls (Figure S2). The pathological sections of the mouse lung tissues were scored, and the results are shown in Figure 1F. It was proved that the A09 strain had the strongest pathogenicity, followed by A01 and A05. There was a significant difference between A05 and A09. Viral titers of H3N8 were further determined in multiple tissues of infected mice (Figure 1G–I). All three infection groups showed detectable viral loads across various organs, with the highest titers consistently observed in the lungs, where viral replication peaked on day 5 post-infection and began to decline by day 7. Although H3N8 virus was detectable in all tissues, in the A05-infected group, a trace amount of virus identified in the brain, with one mouse on day 3 and another on day 5 showing a titer of 10^0.95^ EID_50_/mL. In contrast, mice infected with A01 and A09 displayed more apparent viral loads in the brain, suggesting that these strains possess a certain ability to cross the blood–brain barrier (Figure 1G–I). Meanwhile, A05 has lower titers and reduced dissemination to organs outside of the respiratory tract, which is consistent in Figure 1E,F.

### 3.2. Environmental Persistence of H3N8 Under Diverse Temperature and Surface Conditions

**Figure 2:**
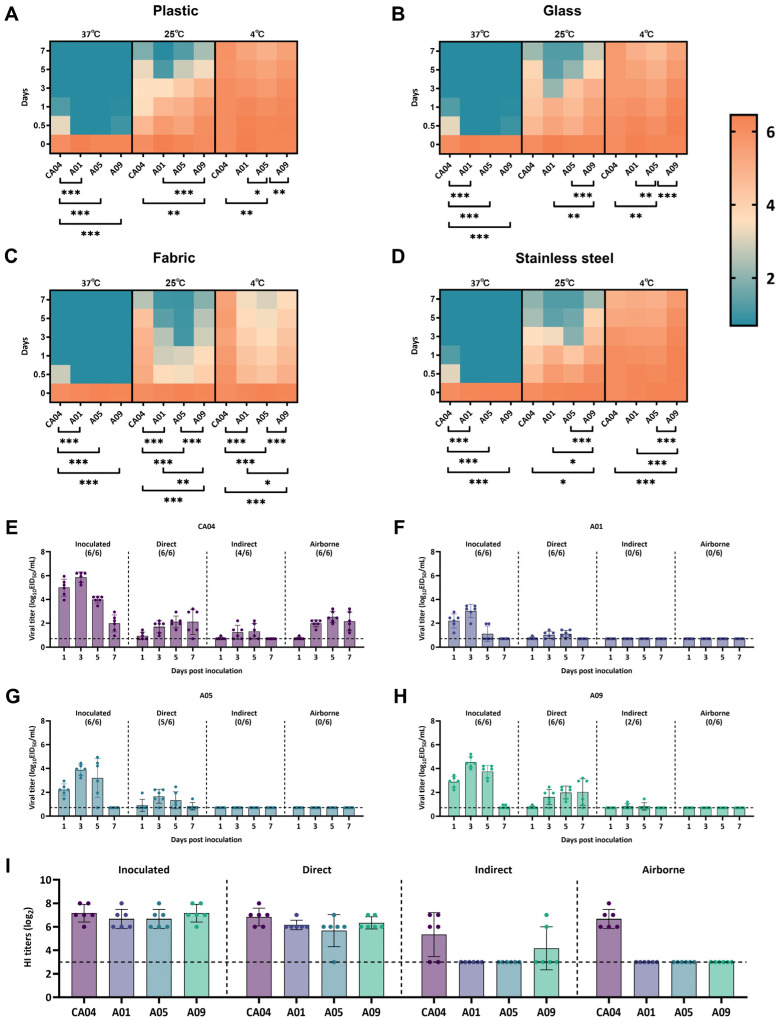
Multi-route transmission efficiency and environmental persistence assess the infection risk of emerging H3N8. (**A**) plastic, (**B**) glass, (**C**) fabric and (**D**) stainless steel represent the surface materials of common items in live poultry markets and were used to test the longest survival time of the virus strain at three temperatures, including 4 °C, 25 °C and 37 °C. * *p* < 0.05, ** *p* < 0.005, *** *p* < 0.0005. (**E**) CA04, (**F**) A01, (**G**) A05 and (**H**) A09 collectively constitute the evaluation of transmission dynamics of emerging H3N8 strains, including direct contact transmission, indirect contact transmission and airborne transmission. Six guinea pigs in donor group were inoculated with influenza virus, and another six guinea pigs in each recipient group were used to conduct transmission experiments. Viral titers of nasal wash samples (**E**–**H**) and HI test of serum (**I**) were used to identify infection. Each data point represents an individual guinea pig.

To further evaluate the environmental transmission risk of H3N8, we assessed its environmental persistence under different temperature and surface conditions (Figure 2A–D). The experiment involved applying each virus to four different materials (fabric, stainless steel, plastic, and glass), followed by incubation at three different temperatures (4 °C, 25 °C, and 37 °C). Measurements were taken at five time points (0.5 h, 1, 3, 5, and 7 days) for each temperature. The human-isolate CA04 was used as the reference strain for transmission (Figure 2E,I) and environmental persistence experiment (Figure 2A–D). The environmental persistence assay results showed that H3N8 viruses remained viable for different durations depending on the surface type and temperature (Figure 2A–D). Overall, viral titers of H3N8 infected group declined more rapidly at 37 °C, with infectivity lost within 1 day on all tested surfaces. Only A09 was detected on the plastic and glass surfaces on the 0.5th day. At 25 °C, viruses exhibited intermediate persistence, persisting for several days, whereas at 4 °C they remained detectable for up to 7 days across all surfaces. Among the surface types, viral persistence was generally longer on smooth materials such as plastic, glass, and stainless steel, while survival was reduced on porous surfaces such as fabric. Strains A09 exhibited greater environmental persistence than the other H3N8 strains across different temperatures and surface types. Notably, under specific conditions, A09 showed significantly higher persistence than the control virus CA04, including on stainless steel at 4 °C and on plastic and glass surfaces at 25 °C. These findings indicate that lower temperatures and smooth, non-porous surfaces favor prolonged persistence of H3N8 viruses in the environment, and the A09 strain is relatively stable under the three temperature conditions.

### 3.3. Multi-Route Transmission Drive H3N8 Infection Risk

Since all three H3N8 strains remained relatively stable at 25 °C, we assessed its transmissibility among guinea pigs through multiple routes was carried out at an ambient temperature of 25 °C. Hartley strain female guinea pigs were used for direct contact, airborne transmission, and indirect contact transmission experiments [22,23]. For direct contact transmission, six donor guinea pigs at 24 h post inoculation were placed together with six naïve guinea pigs [24]. For airborne transmission, additional six naïve guinea pigs were placed in adjacent cages, separated by 5 cm, without direct physical contact with the infected donors [1]. For indirect contact transmission, six donor guinea pigs at 24 h post inoculation were housed in a clean cage, then they were removed after another 24 h and six naïve guinea pigs were subsequently introduced into the same cage to assess transmission through contaminated environments. Nasal wash samples were collected on 1, 3, 5 and 7 days post inoculation. Serum was collected from all guinea pigs 21 days post inoculation and the HI test was performed according to the protocol described in the World Health Organization guidelines. The transmission experiments showed that strain A09 can be transmitted among guinea pigs through direct contact and indirect contact routes (Figure 2H). The probability of direct contact transmission is 100% (6/6), and the probability of indirect contact transmission is 33% (2/6). Both A05 and A01 can only be transmitted between guinea pigs through direct contact, and the probability of direct contact transmission is 83% (5/6) and 100% (6/6), respectively (Figure 2F,G). The results of the serum test were consistent with those of the nasal wash samples test (Figure 2I). The environmental persistence of the three H3N8 strains at 25 °C differed, with A09 exhibiting significantly greater persistence than A01 and A05. Although all three strains remained detectable at 25 °C, the concentration of surviving infectious virus particles was relatively low and insufficient to reach the dose required for 100% infection. Consequently, the indirect transmission efficiency was 0% for A01 and A05, while A09 exhibited an efficiency of 33%. None of the three H3N8 strains can be transmitted among guinea pigs via the airborne route. We further compared the PB2 gene at the key amino acid position 627 [25], which is critical for airborne transmission, among the three H3N8 strains. All three viruses possess E627, with no 627K mutation detected, consistent with their inability to transmit via the airborne route in our guinea pig experiments.

## 4. Conclusion and Discussion

### 4.1. Summary of This Study

In this study, we monitored an environmentally derived H3N8 strain capable of transmission among mammals via indirect contact routes. This strain exhibited robust environmental persistence, which may result in a prolonged half-life of viral particles released into the environment by infected animals. In southern China, where the annual average temperature exceeds 20 °C, the A09 strain can persist in LPM environments for over seven days during most of the year. This durability provides a prerequisite for the cross-species transmission of the H3N8 avian influenza virus to humans. Therefore, it is imperative to implement long-term serological surveillance among workers in LPMs to promptly assess occupational exposure risks and the zoonotic potential of H3N8, thereby enabling timely containment of potential outbreaks. Additionally, these findings reinforce the need for routine environmental biohazard monitoring and disinfection to mitigate zoonotic risk.

### 4.2. Environmental Biohazard Monitoring

Our monitoring results found that H3N8 existed in LPMs environment including chicken feces, cage surface samples, and wastewater, thus establishment of sensitive viable virus detection methods is important for human infection risk assessment [26,27]. Building on these observations and recent advances in environmental virology, high-frequency, real-time monitoring enhances both analytical sensitivity and operational decision speed [28,29,30,31]. When recovery of viable virus is required, water-based condensational growth collectors and other high-efficiency samplers, as well as newer upstream concentration strategies, are preferred to preserve infectivity and maintain downstream assay performance [32,33,34,35]. For nucleic-acid detection, digital polymerase chain reaction (digital PCR) complements conventional PCR at low copy numbers, while viability reverse-transcription quantitative PCR (RT-qPCR) based on capsid-integrity better reflect infectivity after sampling; additionally, CRISPR-Cas9-enriched next-generation sequencing (CRISPR-NGS) boosts sensitivity for low-abundance, highly diverse genetic targets, expanding detection breadth and lowering limits of detection in wastewater surveillance [36,37,38,39,40,41]. For infection risk quantification, quantitative microbial risk assessment (QMRA) based on computational fluid dynamics techniques and dose response models can provide more accurate prediction results that integrated environmental persistence including survival and decay kinetics across temperature, humidity, and surface type [42,43,44]. In addition, robotics, artificial intelligence (AI), and the Internet of Things (IoT) provide real-time monitoring and warning [45,46,47].

### 4.3. Environmental Biohazard Disinfection

Biosafety risk begins with operation, shows with monitoring and ends with management [48,49]. Environmental disinfection is the most common measure, but the current disinfection strategy is often limited to a fixed time, lacking the real-time adjustment ability based on the monitoring results of biological hazards. Policies, such as overnight depopulation, market closures, species segregation, and scheduled cleaning, have repeatedly reduced human infection risk in field settings [50,51,52,53,54,55,56,57,58,59,60,61,62,63]. However, opening a real-time monitoring and disinfection system may block the biological hazards at the first time in the risk budding stage, and minimize the diffusion scope and impact. Disinfection schedule could be timely changed according to the monitoring of virus environmental diffusion inside or outside high biosafety risk facilities [64,65].

### 4.4. Further Outlook: A Risk-to-Response Framework

Our study highlights a potential mechanistic link between viral environmental persistence and transmission efficiency. The environmentally derived H3N8 strain A09 exhibited prolonged persistence across a range of surfaces and temperatures, particularly under cooler conditions and on smooth, non-porous materials. The observed 33.3% indirect transmission efficiency of A09 may be attributable to multiple factors. One possibility is that A09 exhibits greater environmental persistence, allowing this strain to remain infectious in the environment for a longer period and thereby extending the temporal window for transmission. Another explanation is that donors infected with A09 shed higher viral loads than those infected with A01 or A05, resulting in greater environmental contamination and an increased likelihood of successful transmission to the indirect contact guinea pigs. By contrast, other H3N8 strains with lower environmental persistence were restricted to direct contact transmission, suggesting that strain-specific differences in persistence may shape multi-route dissemination patterns. In addition, porous materials accelerate viral inactivation through absorption of moisture, rapid drying, physical entrapment, and alterations of the local microenvironment, whereas smooth, non-porous surfaces provide a relatively stable and moist microenvironment, thereby prolonging viral survival [66]. These findings expand the understanding of how environmental factors can modulate infection risk, supporting the notion that robust viral survival in the environment provides a temporal window for cross-species transmission.

In this study, we used influenza virus stocks diluted in PBS to evaluate viral environmental persistence on porous and non-porous surface materials. Previous studies have examined influenza virus persistence on porous or non-porous surfaces under different liquid media and inoculum volumes, using various vehicles such as milk, PBS, or cell culture medium to dilute the virus [67,68,69,70]. These differences in experimental conditions may contribute to inconsistencies among reported findings [71]. Therefore, our results can only be interpreted as reflecting environmental persistence under the specific conditions employed in this study, and any comparison with other reports should carefully consider the influence of differences in media, volumes, and surface types.

Collectively, our data motivate an evaluation method integrated profiling from genotype to phenotype to predict adaptive roadmap of H3N8 from avian to mammalian host, and an infection risk assessment integrated multi-route transmission efficiency and environmental persistence under multiple key factors. Spread efficiency mainly depends on direct contact, indirect contact and aerosol transmission routes [1,4,64,72]; environmental persistence map usually covers virus viability beyond different materials, temperature and humidity [14,73]; QMRA reveals infection based on computational fluid dynamics techniques and dose response models to perform an overall analysis integrating multiple factors including virus infectivity, transmissibility and environmental persistence [74]. Moreover, these advances highlight the promising potential of intelligent, automated environmental surveillance systems for future applications in public health preparedness.

## Data Availability

The data supporting the findings of this study are available from the corresponding author upon reasonable request.

## Supplementary Materials

The following supporting information can be downloaded at: https://www.mdpi.com/article/10.3390/microorganisms13122782/s1, Figure S1: Replication kinetics of three H3N8 strains in MDCK and A549 cells.; Figure S2: H&E staining of mice lung sections.

## Abbreviations

The following abbreviations are used in this manuscript:

AIV	Avian influenza virus
LPMs	Live poultry markets
ML	Maximum likelihood
MCMC	Markov chain Monte Carlo
MCC	maximum clade credibility
cRBCs	chicken red blood cells
VCNA	Vibrio cholerae neuraminidase
MOI	multiple of infection
EID_50_	Median infective dose
HI	Hemagglutination inhibition
digital PCR	digital polymerase chain reaction
RT-qPCR	reverse-transcription quantitative PCR
CRISPR-NGS	CRISPR-Cas9-enriched next-generation sequencing
QMRA	quantitative microbial risk assessment
AI	artificial intelligence
IoT	Internet of Things
