# Supplementary material for: From Surfaces to Spillover: Environmental Persistence and Indirect Transmission of Influenza A(H3N8) Virus
Source: Microorganisms. 2025 Dec 6;13(12):2782. doi: 10.3390/microorganisms13122782 (PMC12735991; doi:10.3390/microorganisms13122782)
Supplement: Supplementary file 1 [file microorganisms-13-02782-s001.zip › microorganisms-4004869-supplementary.pdf]

Supplementary Materials

From Surfaces to Spillover: Environmental Persistence and In-direct Transmission of Influenza A(H3N8) Virus

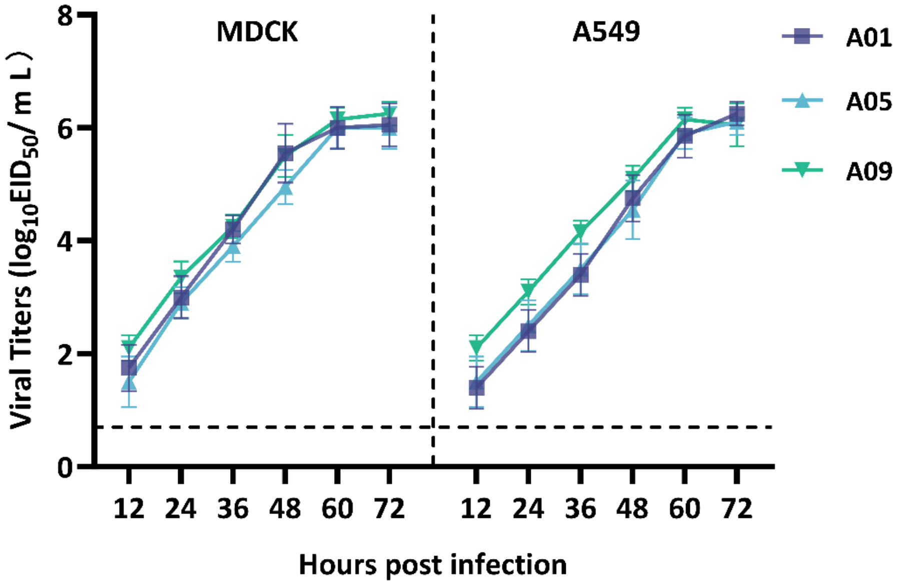

FIGURE S1. Replication kinetics of three H3N8 strains in MDCK and A549 cells.

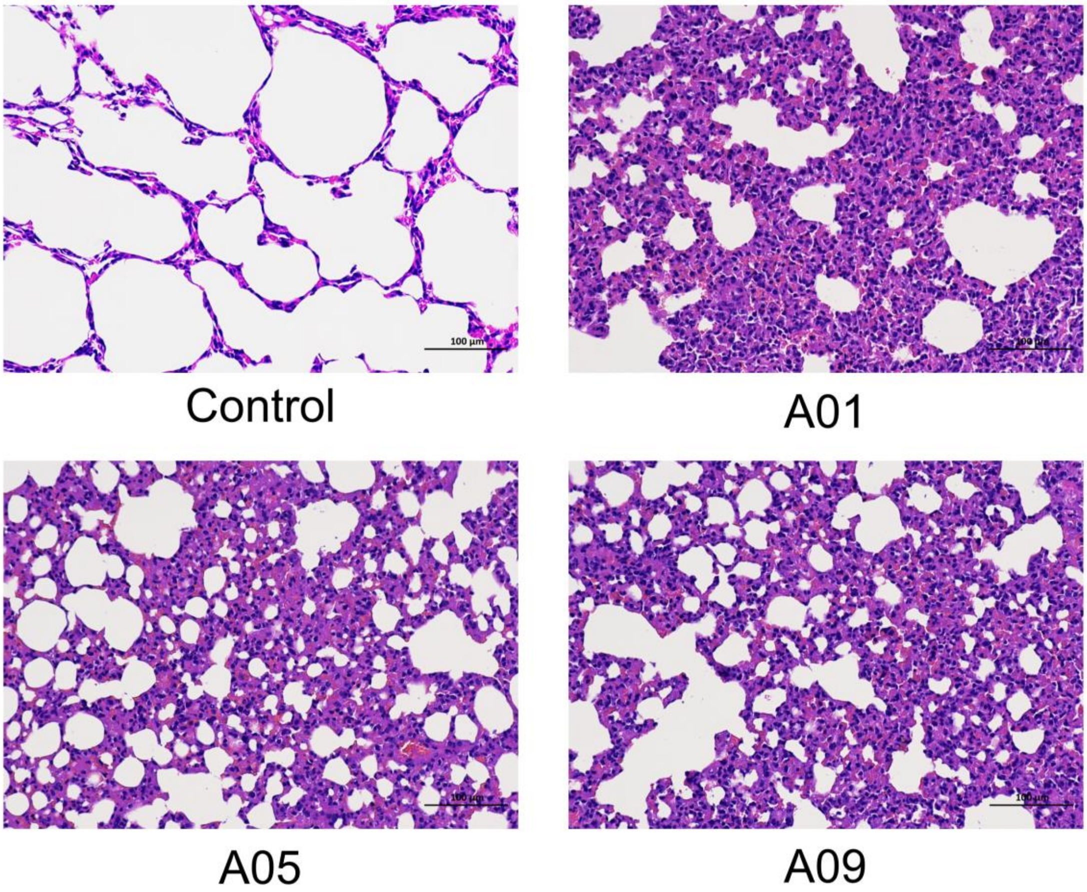

FIGURE S2. H&E staining of mice lung sections.
